# Recent Progress in the Voltage-Controlled Magnetic Anisotropy Effect and the Challenges Faced in Developing Voltage-Torque MRAM

**DOI:** 10.3390/mi10050327

**Published:** 2019-05-15

**Authors:** Takayuki Nozaki, Tatsuya Yamamoto, Shinji Miwa, Masahito Tsujikawa, Masafumi Shirai, Shinji Yuasa, Yoshishige Suzuki

**Affiliations:** 1 National Institute of Advanced Industrial Science and Technology (AIST), Spintronics Research Center, Tsukuba, Ibaraki 305-8568, Japan; yamamoto-t@aist.go.jp (T.Y.); yuasa-s@aist.go.jp (S.Y.); suzuki-y@mp.es.osaka-u.ac.jp (Y.S.); 2 The Institute of Solid State Physics, The University of Tokyo, Kashiwa, Chiba 277-8531, Japan; miwa@issp.u-tokyo.ac.jp; 3 Graduate School of Engineering Science, Osaka University, Toyonaka, Osaka 560-8531, Japan; 4 Research Institute of Electrical Communication, Tohoku University, Sendai, Miyagi 980-8577, Japan; t-masa@riec.tohoku.ac.jp (M.T.); shirai@riec.tohoku.ac.jp (M.S.)

**Keywords:** voltage-controlled magnetic anisotropy, magnetoresistive random access memory, magnetic tunnel junction

## Abstract

The electron spin degree of freedom can provide the functionality of “nonvolatility” in electronic devices. For example, magnetoresistive random access memory (MRAM) is expected as an ideal nonvolatile working memory, with high speed response, high write endurance, and good compatibility with complementary metal-oxide-semiconductor (CMOS) technologies. However, a challenging technical issue is to reduce the operating power. With the present technology, an electrical current is required to control the direction and dynamics of the spin. This consumes high energy when compared with electric-field controlled devices, such as those that are used in the semiconductor industry. A novel approach to overcome this problem is to use the voltage-controlled magnetic anisotropy (VCMA) effect, which draws attention to the development of a new type of MRAM that is controlled by voltage (voltage-torque MRAM). This paper reviews recent progress in experimental demonstrations of the VCMA effect. First, we present an overview of the early experimental observations of the VCMA effect in all-solid state devices, and follow this with an introduction of the concept of the voltage-induced dynamic switching technique. Subsequently, we describe recent progress in understanding of physical origin of the VCMA effect. Finally, new materials research to realize a highly-efficient VCMA effect and the verification of reliable voltage-induced dynamic switching with a low write error rate are introduced, followed by a discussion of the technical challenges that will be encountered in the future development of voltage-torque MRAM.

## 1. Introduction

The evolving information society has triggered the rapid spread of advanced technologies, such as Artificial Intelligence (AI), Advanced Safety Vehicle (ASV), and IoT (Internet of Things), and this has led to further industrial innovation. In the society of the future, Big-Data collected from physical space will be stored and analyzed in cyber space, which creates new social values. Such a data-driven society can only be sustained by the high-speed processing of Big-Data; therefore, reducing the power consumption of nano-electronic devices is becoming increasingly crucial. One promising approach is the introduction of nonvolatile computation.

It is expected that the stand-by power of future computing systems will be reduced by utilizing the nonvolatile features of spintronic devices, such as a magnetoresistive random-access memory (MRAM) while using magnetic tunnel junctions (MTJ). An MTJ consists of two ferromagnetic layers that are separated by an ultrathin insulating layer, such as magnesium oxide (MgO) [1,2]. Electrons can tunnel through the barrier when a bias voltage is applied between the two ferromagnetic layers due to the ultrathin thickness of the insulating layer. The amplitude of the tunneling current depends on the relative angle between the magnetizations in each ferromagnetic layer through a spin-dependent tunneling process, which is called the tunnel magnetoresistance (TMR) effect. The direction of the magnetizations of one of the ferromagnetic layers is fixed (reference layer), typically by exchange coupling with an antiferromagnetic material. An external field (free layer), using an electric-current, can control the direction in the other, as discussed below. In this way information is written to the memory device. Then, the information can be stored by controlling the magnetization configuration between parallel and anti-parallel states, exhibiting two resistance states, in a nonvolatile manner.

MRAM has great potential to be a fast, high write endurance, and CMOS-compatible nonvolatile memory, which is suitable for embedded as well as standalone memory applications. However, one of the significant remaining challenges is to reduce the energy that is needed to write information, that is, to switch the magnetization. In the long history of magnetism, magnetic fields that are produced by electric-current have been used for magnetization reversal. This indirect approach is inefficient and not scalable. Spintronics has brought us a new way of switching the magnetization through the *s-d* exchange interaction between the conduction electron spin and localized spin, called the spin-transfer torque (STT) effect [3,4,5,6,7,8]. The spin angular momentum that is carried by conduction electrons can be transferred to localized electrons and can induce magnetization reversal. Recently, an alternative technique for magnetization switching using the spin Hall effect, which is called the spin-orbit torque (SOT) switching [9,10,11,12], has also been attracting attention. A typical SOT device comprises a bilayer that consists of a non-magnetic heavy metal layer, such as Ta or W, and a ferromagnetic layer capped by an oxide. A transverse pure-spin current is generated when an in-plane electric-current is injected into the bilayer due to the spin Hall effect. The accumulation of spin at the heavy metal/ferromagnet interface exerts a torque and induces magnetization switching. In this switching scheme, high write endurance can be realized, even with high speed switching of the order of a few nanoseconds, because the read and write passes are separate. 

**Figure 1:**
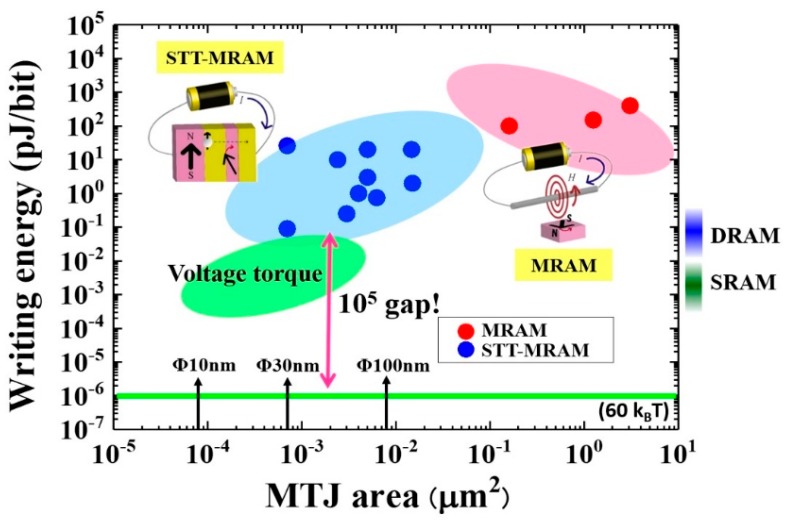
Reported writing energy for toggle magnetoresistive random-access memory (MRAM) (red dots) and spin-transfer torque-based switching (STT-MRAM) (blue dots) as a function of magnetic tunnel junctions (MTJ) cell size and the target area for voltage-torque MRAM.

STT-based switching (STT-MRAM) has brought a drastic reduction in writing energy and expanded potential for applications; STT-MRAM [13,14,15]. Figure 1 summarizes the reported writing energies for a MRAM (red dots) and STT-MRAM (blue dots) as a function of the MTJ cell size. For example, recent developments in STT-MRAMs have achieved writing energies of approximately 100 fJ/bit in perpendicularly magnetized MTJs [13], which is close to the writing energy for a dynamic-RAM (DRAM). However, it is still much higher than that of a static-RAM (SRAM), which is made up of several MOSFETs that an electric-field operates. Furthermore, a writing energy of 100 fJ/bit corresponds to 10^7^ *k*_B_*T* (*k*_B_ is the Boltzmann constant and *T* is the temperature, assumed to be 300 K). On the other hand, the energy that is required to maintain magnetic information, i.e. the thermal stability, is about 60 *k*_B_*T* (green line in Figure 1), which means that we have a large energy gap between data writing and retention, in the order of 10^5^. This difference mainly comes from unwanted energy consumption due to ohmic dissipation of the electric-current flow. Overcoming this fundamental issue using a novel way of electric-field based spin manipulation is strongly desired. Not only for MRAMs, but all of the nonvolatile memories that have been proposed so far have a dilemma of choosing between stable nonvolatility and high operating energy. Therefore, the development of a novel type of memory having low operating energy as well as low stand-by energy can have great impact on the design of future memory hierarchy.

Various kinds of approaches to the electric-field manipulation of spin have been proposed and experimentally demonstrated, such as using the inverse magnetostriction effect in a multilayered stack with piezoelectric materials [16,17,18], the gate-controlled Curie temperature in ferromagnetic semiconductors [19,20,21] or even in an ultrathin ferromagnetic metal layer [22], magnetoelectric switching of exchange bias [23,24,25,26], electric polarization induced control in magnetic anisotropy at the ferromagnetic/ferroelectric interface [27,28], electric-field induced magnetic phase transition through structural phase transition [29], and the utilization of multiferroic materials [30,31]. However, each of these approaches have the drawbacks of limited operation temperature or low write endurance or difficulty in the introduction to magnetoresistive devices, although these requirements should be simultaneously satisfied for memory applications. We have focused on the voltage-controlled magnetic anisotropy (VCMA) effect in an ultrathin ferromagnetic layer [32,33] to overcome this problem. 

This paper reviews recent progress in the research of the VCMA effect and the challenges that are faced in developing new types of MRAM controlled by voltage, called voltage-torque MRAM (also called Magnetoelectric (ME)-RAM) [34,35,36,37,38,39]. Section 2 presents an overview of the early experimental observations of the VCMA effect in all-solid state devices and the concept of voltage-induced dynamic switching, with a discussion of the technical challenges. In Section 3, the current understanding of the physical origin of the VCMA effect is discussed through experimental investigations while using X-ray absorption spectroscopy (XAS) and magnetic circular dichroism (XMCD) analyses with first-principles calculation. Section 4 presents the materials research being done to enhance the VCMA effect, especially focusing on the heavy metal doping technique. Finally, in Section 5, experimental demonstrations of reliable voltage-induced dynamic switching and an understanding of the voltage-induced spin dynamics are discussed, together with a discussion on the theoretical investigations being made.

## 2. Overview of the VCMA Effect and Voltage-Induced Dynamic Switching

Weisheit et al. first reported the VCMA effect in a 3*d* transition ferromagnetic layer in 2007 [32]. They observed a coercivity change of a few % in 2–4 nm-thick FePt(Pd) films immersed in a liquid electrolyte. Opposing trends in the change in coercivity in FePt and FePd, depending on the applied voltage, were observed. An electric double layer is effective for applying a large electric-field at the interface; however, the operating speed is limited and we need to take care of the influence of chemical reactions. The voltage control of in-plane magnetic anisotropy was also found in ferromagnetic semiconductors at low temperature [40]. Theoretical attempts to understand the physical origin of the VCMA effect in metal started around the same time. Duan et al. proposed that spin-dependent screening of the electric-field can induce modification in the surface magnetization and magnetic anisotropy [41]. Nakamura et al. calculated the VCMA effect in a freestanding Fe(001) monolayer and pointed out that electric-field induced changes in the band structure, especially the *p* orbitals near the Fermi level, which are coupled to the *d* states, play an important role [42]. Tsujikawa et al. studied the VCMA effect in a Pt/Fe/Pt/vacuum system and found that relative modification in the electron filling of the 3*d* orbital induced by the accumulated charges at the interface causes a change in the perpendicular magnetic anisotropy (PMA) [43]. Other possible mechanisms have also been discussed, such as electric-field induced modification in Rashba spin-orbit anisotropy [44,45] and electric-field induced atomic displacement at the interface between ferromagnetic oxide and dielectric layers [46].

**Figure 2:**
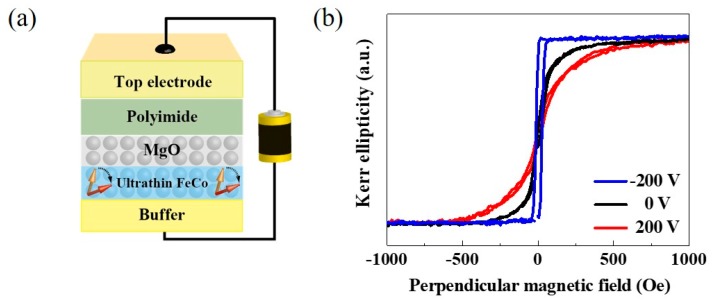
(**a**) Schematic illustration of sample stack used for the first demonstration of the voltage-controlled magnetic anisotropy (VCMA) effect in an all-solid state structure, and (**b**) applied bias voltage dependence of the polar-magneto-optical Kerr effect (MOKE) hysteresis curves for a 0.58 nm-thick Fe_80_Co_20_ layer.

We attempted to apply the VCMA effect in an all solid state structure, which consisted of epitaxial Au/ultrathin Fe(Co)/MgO/polyimide/ITO junctions grown on MgO(001) substrates (see Figure 2a) to investigate the feasibility for practical applications [33,47]. Figure 2b shows an example of polar magneto-optical Kerr effect (MOKE) hysteresis curves that were measured under the application of a voltage. The thickness of the Fe_80_Co_20_ layer is fixed at 0.58 nm. The bias direction is defined with respect to the top ITO electrode. A clear change in the saturation field in the out-of-plane direction can be seen, which suggests a modification in the PMA. Under the application of a positive bias, the PMA is suppressed and the in-plane anisotropy becomes more stable. On the other hand, the application of a negative voltage enhances the PMA and even the transition of the magnetic easy axis can be realized from the in-plane to the out-of-plane direction.

Due to screening by free electrons, the penetration of the electric-field into a metal is limited to the surface, unlike in the case of a semiconductor; however, if the thickness of the ferromagnetic layer is thin enough, e.g. several monoatomic layers, the modulation in the electronic structure at the interface can make a sizable impact on the magnetic properties. Details of an experimental verification for the physical origin of the VCMA effect are discussed in Section 2.

**Figure 3:**
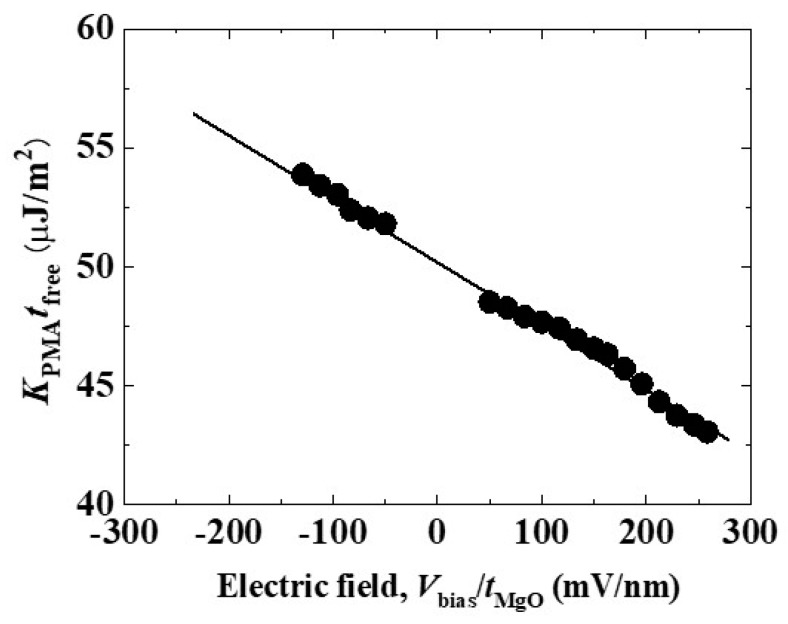
Example of applied electric-field dependence of *K*_PMA_*t*_free_ observed in an MgO-based MTJ structure. Reprinted figure with permission from [48], Copyright 2010 by the AIP Publishing LLC.

One great advantage of the VCMA effect is its high applicability in a MTJ structure, which is the most important practical devices in spintronics. Figure 3 exhibits the first demonstration of the VCMA effect that was observed in a MTJ structure, which consisted of Cr/ Au/ultrathin Fe_80_Co_20_(0.5 nm)/MgO(*t*_MgO_)/Fe grown on a MgO(001) substrate [48]. Here, we made electrical ferromagnetic resonance (FMR) measurements through the TMR effect. The PMA energy, *K*_PMA_, was evaluated from the resonant frequency of the free layer at each applied voltage. In addition to FMR measurements, the effect of a bias voltage on normalized TMR curves has also often been used for the quantitative evaluation of the VCMA effect, as discussed later [49]. Generally, the PMA energy linearly changes as a function of the applied electric field, *E*, which is defined as the applied bias voltage, *V*_bias_, divided by the MgO thickness, *t*_MgO_. The slope of the linear relationship represents the VCMA coefficient in units of J/Vm, *e.g.* −37 fJ/Vm for the case in Figure 3. The VCMA coefficient is one of the most important parameters for demonstrating scalability and also in the reliable switching of the magnetization and thus the development of voltage-torque MRAM.

The realization of the VCMA effect in all-solid state devices, including a MTJ structure, made it possible for us to demonstrate the high speed response of this effect, such as in voltage-induced ferromagnetic resonance excitation [50,51,52,53,54], dynamic magnetization switching driven solely by the application of a voltage [55], and spin wave excitation [56,57,58].

In addition to ultrathin epitaxial films with large PMA [35,59,60,61,62,63,64,65,66,67], VCMA effects have been observed in various materials systems, for example, in sputter-deposited CoFeB [68,69,70,71,72,73,74,75,76,77,78,79,80,81], which is an important practical material that is used in the mass production of MTJs, and in self-assembled nano-islands [82], nanocomposite structures [83], and ultrathin layers with quantum well states [84]. The VCMA effect can also be applied for the control of domain wall motion [85,86,87] and magnetic skyrmions [88,89,90]. In addition, voltage control of the magnetic properties has been expanded not only for the PMA, but also for the Curie temperature [22], Dzyaloshinskii-Moriya interactions [91], interlayer exchange coupling [92], and proximity-induced magnetism in non-magnetic metal thin films [93,94,95].

The VCMA effect can induce a transition of the magnetic easy axis between the in-plane and out-of-plane directions by the application of a static voltage; however, bi-stable switching is not easily attained, because the VCMA effect does not break the time reversal symmetry. One possible way is to use the VCMA effect to assist other external fields. For example, the coercivity of the perpendicularly magnetized film can be reduced by the application of dc voltage [47,96,97] or of voltage-induced FMR [98], just as in the microwave-assisted magnetization reversal (MAMR) technique. Moreover, the combination of STT [99,100] or SOT [101] and the VCMA effect has also been experimentally demonstrated. These approaches are effective in reducing the energy that is required for writing by electric-current based manipulation; however, the realization of magnetization switching solely by a voltage effect is much more preferable.

**Figure 4:**
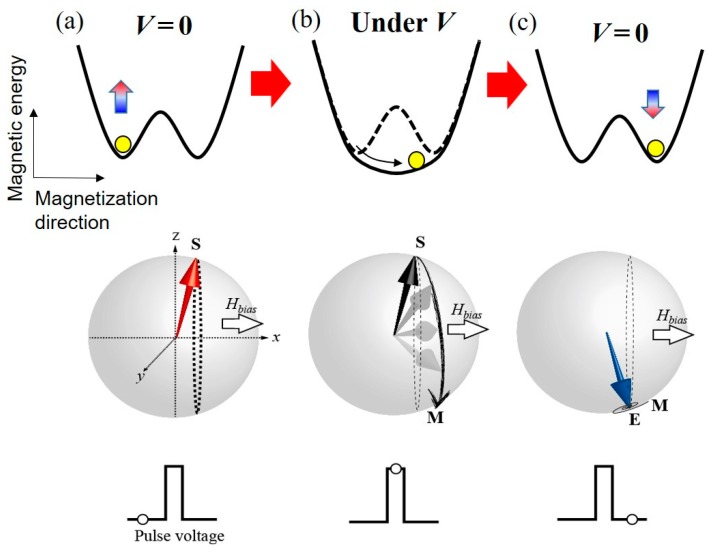
Conceptual diagram of voltage-induced dynamic switching for a perpendicularly-magnetized film. The in-plane bias magnetic field, *H*_bias_, which determines the axis of the precessional dynamics, is applied in the +*x* direction. (**a**) initial state (point S), (**b**) precessional switching process induced by an application of pulse voltage (from point S to point M), and (**c**) relaxation process (from point M to point E).

We proposed pulse voltage-induced dynamic switching to overcome this problem (see Figure 4). This technique was first demonstrated in in-plane magnetized MTJs [55,102] and it was then applied in perpendicularly-magnetized MTJs [103,104,105,106,107,108,109]. For example, we assume the initial state (Figure 4a) to be the perpendicularly magnetized “up” state under the application of an in-plane bias magnetic field, *H*_bias_. When a short pulse voltage is applied to eliminate the PMA completely, the magnetization starts to precess around the *H*_bias_ (Figure 4b). If the voltage pulse is turned off at the timing of half turn precession, then the magnetization can be stabilized in the opposite “down” direction (Figure 4c). *H*_bias_ is required to determine the axis of magnetization precession. The effective field, such as crystalline anisotropy field and the exchange bias field, is also applicable.

**Figure 5:**
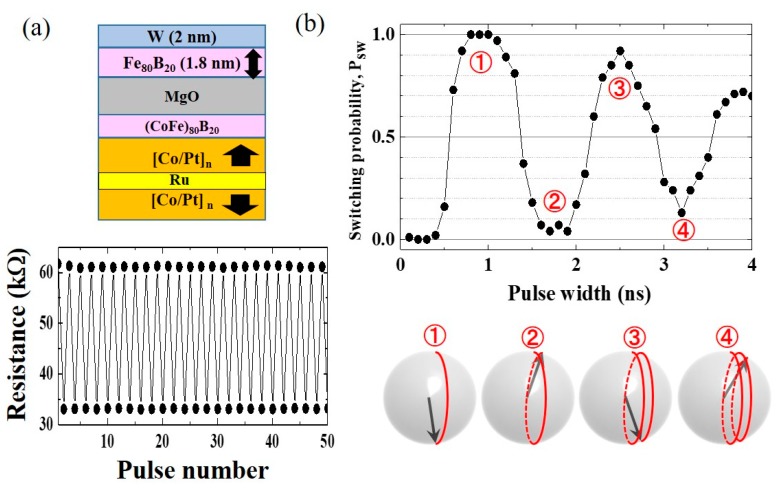
Experimental demonstration of voltage-induced dynamic switching. (**a**) Schematic of the sample structure of a voltage-controlled perpendicularly-magnetized MTJ and observed bi-stable switching between parallel and antiparallel magnetization configurations induced by successive pulse voltage applications. (**b**) Pulse width dependence of switching probability, *P*_SW_. Due to the precessional dynamics, *P*_SW_ exhibits oscillatory behavior depending on the pulse width.

Figure 5a shows an example of an experimental demonstration of voltage-induced dynamic switching being observed in perpendicularly magnetized MTJs [105]. The top FeB layer with a W cap is the voltage-driven free layer. Under an optimized applied magnetic field, we achieved the stable toggle switching by the successive application of voltage pulses with a width of 1 ns and amplitude of −1.2 V. The precessional dynamics of the magnetization are reflected in the oscillation of the switching probability (*P*_SW_) as a function of pulse width, as shown in Figure 5b. A high *P*_SW_ is obtained at the timing of half turn precession; however, when the pulse width is twice this, one turn precession results in low *P*_SW_. From a practical point of view, the first half turn precession is effective in obtaining a low WER with fast switching speed. Under the condition that the PMA is completely eliminated, the amplitude of *H*_bias_ determines the precession frequency, and then the switching time, *t*_SW_ for the half turn precession is expressed as

(1) $$t_{\mathrm{SW}}\sim \frac{\pi \left(1-\alpha^{2}\right)}{\gamma \mu_{0}H_{\mathrm{bias}}}$$

where *α*, *γ*, and *μ*_0_ are the magnetic damping constant, the gyromagnetic ratio, and the permeability of vacuum, respectively.

The possible advantages of voltage-induced dynamic switching are as follows. (ⅰ) Fast switching ( ~1 nanosecond) can be induced with an ultralow switching power of the order of a few fJ/bit. (ⅱ) The switching transistor can be downsized, because we do not need to apply a large electric-current. (ⅲ) Unipolar switching can separate the polarity of voltages for writing and reading. In addition, the VCMA-induced enhancement in PMA has been used to propose a unique approach to reduce the read disturbance [110].

On the other hand, the following technical challenges remain. Firstly, the realization of a large VCMA effect is the most important issue to show the scalability of the voltage-torque MRAM, as discussed in Section 4. Furthermore, as seen in Figure 5b, the switching probability is sensitive to the writing pulse width, due to the precession-mediated switching process. Therefore, we need verification as to whether a sufficiently-low WER can be achieved by the voltage-induced dynamic switching technique. In addition, this is a toggle switching technique, so pre-read and read-verify processes are always required for writing. These reading processes dominate the total write time, and it can be critical when the resistance of the MTJ cell increases. In addition, the removal of the external magnetic field is also an important issue for practical applications.

## 3. Physical Origin of the VCMA Effect

**Figure 6:**
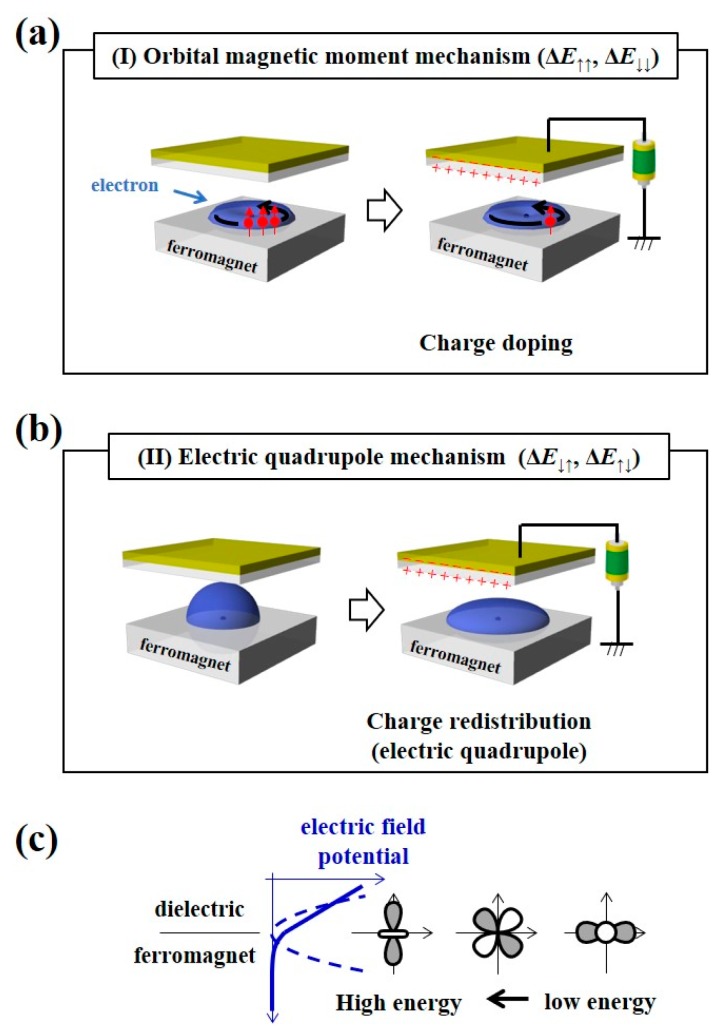
Microscopic origin of the VCMA effect. (**a**) Orbital magnetic moment mechanism. (**b**) Electric quadrupole mechanism. (**c**) Schematic of the nonlinear electric field at the interface between the dielectrics and the ferromagnet, which induces a charge redistribution-induced VCMA effect.

In this section, recent experimental trials conducted to understand the physical origin of the VCMA effect are introduced [111]. The following two mechanisms account for the purely electronic VCMA effect. The first mechanism comes from the charge-doping-induced anisotropy in the orbital angular momentum, as shown in Figure 6a. As each electron orbital in the vicinity of the Fermi level has a different density of states, selective charge doping may induce anisotropy in the orbital angular momentum. This effect changes the PMA energy through spin-orbit interactions from the spin-conserved virtual excitation processes [112,113], as expressed by the first term in Equation (2) [114].

(2) $$-\frac{1}{4}\frac{\lambda }{\hbar }\left(\langle \Delta L_{\xi ,\;↓↓}\rangle -\langle \Delta L_{\xi ,\;↑↑}\rangle \right)+\frac{7}{2}\frac{\lambda }{\hbar }\left(\langle \Delta T_{\zeta ,\;↓↑}^{\prime }\rangle -\langle \Delta T_{\zeta ,\;↑↓}^{\prime }\rangle \right)$$

Here, *λ* is the spin-orbit interaction coefficient. *L* and *T′* are the orbital angular momentum and part of the magnetic dipole operator, respectively. Here, $\langle \Delta L_{\xi }\rangle \equiv \langle L_{z}\rangle -\langle L_{x}\rangle$ and $\langle \Delta T_{\zeta }^{\prime }\rangle \equiv \langle T_{z}^{\prime }\rangle -\langle T_{x}^{\prime }\rangle$ are used. $\langle L_{z}\rangle$ and $\langle L_{x}\rangle$ are evaluated for the *z*- and *x*- components of the spin angular momentum, respectively. The same is the case for $\langle T_{z}^{\prime }\rangle$ and $\langle T_{x}^{\prime }\rangle$. ↑ and ↓ denote the contributions from the majority and minority spin-bands, respectively. We call the first mechanism the orbital magnetic moment mechanism. The second mechanism is the VCMA effect from the induction of an electric quadrupole (Figure 6b). An electric-field applied to the metal/dielectric interface is inhomogeneous, owing to the strong electrostatic screening effect in the metal, such as electric-field, including higher-order quadratic components, can couple with the electric quadrupole correlated with the magnetic dipole operator in an electron shell of the metal layer. The induced energy split of each orbital changes the magnetic anisotropy through spin-orbit interactions from spin-flip virtual excitation processes [115,116], as shown in Figure 6c. The latter mechanism corresponds to the second term in Equation (2). We call this the electric quadrupole mechanism. As the expectation values for the orbital angular momentum and the magnetic dipole operator can be measured as the orbital magnetic moment and the magnetic dipole *T_z_* term (*m*_T_), respectively, the aforementioned two mechanisms can be validated by X-ray absorption spectroscopy (XAS) and X-ray magnetic circular dichroism (XMCD) spectroscopy.

**Figure 7:**
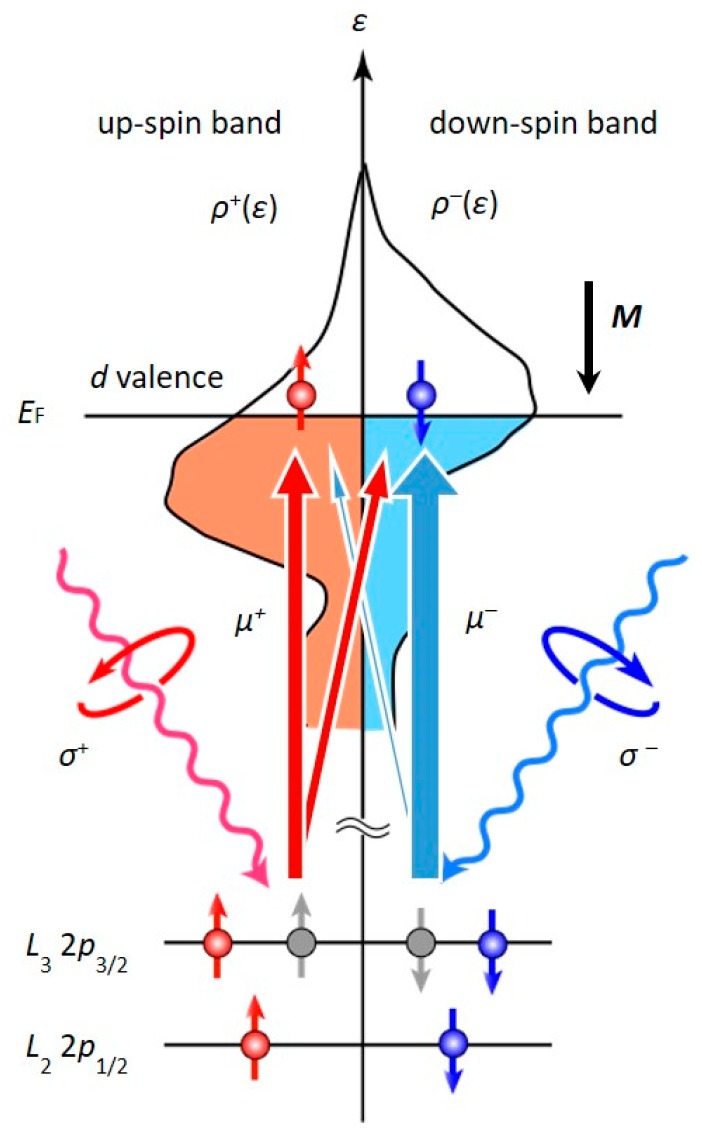
Diagram of the electronic states related to X-ray absorption spectroscopy and X-ray magnetic circular dichroism (XAS/XMCD) measurements at the *L*-edges of transition metals.

The XAS/XMCD experiments provide element-specific information on the electronic structure via the optical transition from the core level to unoccupied states in the valence band. Based on the use of circularly polarized X-rays, X-ray absorption techniques provide interesting features for the study of magnetic materials. Figure 7 shows a schematic diagram of the electronic states that are involved in an optical transition from the 2*p* core to *d* valence states, which is related to XMCD at the *L* edges of transition metals. The dichroic signal directly reflects the difference in the density of the states near the Fermi level between the up and down spin sub-bands. From the XMCD results with sum-rule analysis [117,118], the magnetic moments (spin magnetic moment: *m*_S_, *m*_L_, and *m*_T_) can be determined from the measured XAS/XMCD spectra. Here, the measured orbital magnetic moments and magnetic dipole *T_z_* term have the following relationships;

(3) $$\begin{array}{c} \Delta m_{L}=-\frac{\mu_{B}\left(\langle \Delta L_{↓↓}\rangle +\langle \Delta L_{↓↓}\rangle \right)}{\hbar },\;\mathrm{and}\\ -7\Delta m_{T}=-\mu_{B}\left(\langle \Delta L_{↑↑}^{2}\rangle -\langle \Delta L_{↓↓}^{2}\rangle \right)-7\mu_{B}\left(\langle \Delta T_{↓↑}\rangle +\langle \Delta T_{↑↓}\rangle \right)/\hbar \end{array}$$

It should be noted that the PMA energy from the spin-conserved virtual excitation process (first term in Equation (2)) is related to the orbital magnetic moment and the PMA energy from the spin-flip virtual excitation process (second term in Equation (2)) is related to the magnetic dipole *T_z_* term.

**Figure 8:**
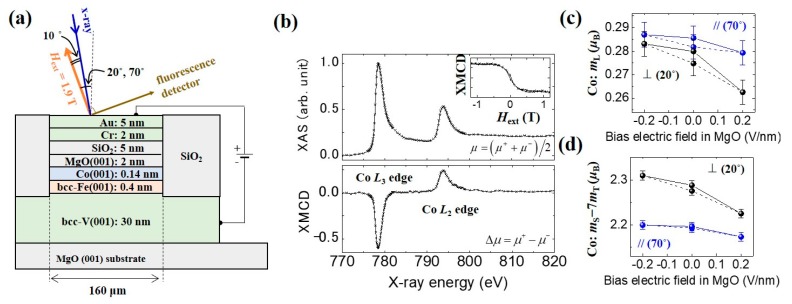
Voltage-induced changes to the magnetic moment of Co in the Fe/Co/MgO system. (**a**) Schematic of the sample structure. (**b**) Typical XAS/XMCD results around the Co-absorption edges. (**c**) Voltage-induced change to the orbital magnetic moment in Co. (**d**) Voltage-induced changes to the effective spin magnetic moment (*m*_S_ − 7*m*_T_) in Co. Reprinted figure with permission from [113], Copyright 2017 by the American Physical Society.

A Fe/Co (1 ML)/MgO multilayer was employed to see the changes in the orbital magnetic moment in XAS/XMCD experiments [113]. The sample stack is depicted in Figure 8a. A multilayered structure, consisting of bcc-V(001) (30 nm)/bcc-Fe(001) (0.4 nm)/Co (0.14 nm)/MgO(001) (2 nm)/SiO_2_ (5 nm)/Cr (2 nm)/Au (5 nm), was deposited on a MgO(001) substrate. Figure 8b shows the typical XAS/XMCD results around the *L*_3_ and *L*_2_ edges of Co with a magnetic field of 1.9 T (*θ* = 20°) to saturate the magnetization of the Fe/Co layer. The changes in the orbital magnetic moment and effective spin magnetic moment (*m*_S_ − 7*m*_T_) of Co were determined while using sum-rule analysis, and they are summarized in Figure 8c,d. We can see that *m*_L_ of Co with an electric-field of −0.2 V/nm is larger than that corresponding to +0.2 V/nm. Moreover, the induced change in *m*_L_ with *θ* = 20° is larger than that with *θ* = 70°. The experiment demonstrates that an orbital magnetic moment anisotropy change of (0.013 ± 0.008)*μ*_B_ between the magnetization angles of *θ* = 20° and 70° was generated in the presence of applied electric fields of ±0.2 V/nm. Figure 8d shows the electric-field-induced change in *m*_S_ − 7*m*_T_ of Co. As with *m*_L_, *m*_S_ − 7*m*_T_ is enhanced under the application of a negative electric-field. Moreover, the electric-field-induced change in the magnetic moment is anisotropic. In contrast to *m*_T_, it is known that *m*_S_ is not sensitive to the magnetization direction. Hence, the anisotropic part of the induced change in the magnetic moment should be attributed to *m*_T_. 

As discussed in the previous section, Equation (2) can be used to analyze the VCMA effect. If we employ the spin-orbit interaction coefficient of Co, *λ*_Co_ = 5 meV, then the induced change in the PMA energy is estimated to be 0.039 ± 0.023 mJ/m^2^ when the applied electric-field is switched from +0.2 V/nm to −0.2 V/nm. Here, the experimentally obtained Δ*m*_L_ = (0.017±0.010)*μ*_B_ was used. From the VCMA coefficient in the Fe/Co/MgO system (−82 fJ/Vm), the PMA energy change at ±0.2 V/nm is 0.03 mJ/m^2^, which is in good agreement with the PMA energy change that was obtained using the first term of Equation (2). From the discussion above, the change in the orbital magnetic moment anisotropy in Co seems to explain the VCMA effect. However, the impact of the change in the magnetic dipole *T_z_* term (*m*_T_) that is shown in Figure 8d on the VCMA effect remains to be seen. In Ref. 113, a first principles study was employed to clarify this point. As a result, the VCMA effect from the spin-flip terms (Δ*E*_↓↑_ + Δ*E*_↑↓_) is found to be negligible and that from the spin-conserved terms (Δ*E*_↑↑_ + Δ*E*_↓↓_) appeared to be dominant. Therefore, the change in orbital magnetic moment is responsible for the VCMA effect. Due to the large exchange splitting for Co, the observed changes in *m*_T_ do not contribute to the VCMA effect, as described by the second term in Equation (2).

**Figure 9:**
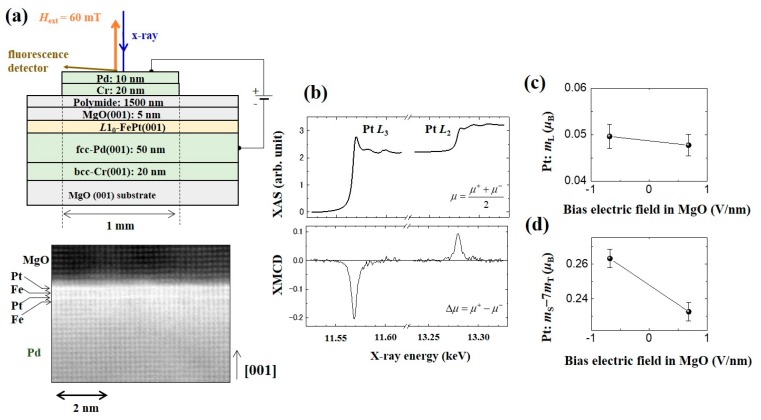
Voltage-induced changes to the magnetic moment of Pt in the Fe/Pt/MgO system. (**a**) Schematic of the sample structure and its high-angle annular dark-field scanning transmission electron microscopy (HAADF-STEM) image. (**b**) Typical XAS/XMCD results around the Pt-absorption edges. (**c**) Voltage-induced change to the orbital magnetic moment in Pt. (**d**) Voltage-induced changes to the effective spin magnetic moment (*m*_S_ − 7*m*_T_) in Pt. Reproduced from [116]. CC BY 4.0.

It has been reported that the spin-orbit interaction energy from a spin-flip virtual excitation process makes a significant contribution to the VCMA effect when 3*d*/5*d*-layered transition metals are employed [116]. Figure 9a shows an experimental design and a high-angle annular dark-field scanning transmission electron microscopy (HAADF-STEM) image of the device. Figure 9b shows the typical results of the polarization-averaged XAS and its XMCD around the *L*_3_ and *L*_2_ energy edges of Pt. A perpendicular magnetic field of ±60 mT was applied to saturate the magnetization of FePt. Figure 9c,d show electric-field-induced changes in the magnetic moments of Pt. The results confirm a clear bias voltage inductions of *m*_S_ − 7*m*_T_, while there is no significant change to *m*_L_ under voltage applications.

In general, in low-symmetry systems, such as interfaces, the atomic electron orbital may possess an electric quadrupole moment. If the atom is also spin-polarized, the electric quadrupole moment induces the anisotropic part of the spin-density distribution, i.e., the magnetic dipole *T*_z_ term (*m*_T_) [114,115,116,118]. In contrast to *m*_T_, *m*_S_ is not sensitive to the magnetization direction. In Ref. 116, the voltage-induced change in *m*_S_ − 7*m*_T_ shows large magnetization direction dependence. Thus, the observations indicate the significant induction of *m*_T_ in Pt by an external voltage. A first-principles study was also conducted for the FePt/MgO system, similar to the Fe/Co/MgO study. As a result, firstly, the monoatomic Pt layer at the interface with MgO makes the dominant contribution to the VCMA effect. Moreover, while the VCMA effect from the spin-conserved terms (Δ*E*_↑↑_ + Δ*E*_↓↓_) decreases the PMA energy, the VCMA effect that is induced by the applied voltage from the spin-flip terms of interfacial Pt increases the PMA energy (Δ*E*_↓↑_ + Δ*E*_↑↓_). The total PMA energy in the FePt/MgO system increases under the condition of electron depletion at the Pt/MgO interface, as the PMA energy increase by the spin-flip terms is greater than the PMA energy decrease by the spin-conserved terms.

To conclude, for the 3*d*-transition ferromagnetic metals, it is important to consider the orbital magnetic moment anisotropy. The validity of the Bruno model [112] (first term of Equation (2) and Figure 6a) has been experimentally demonstrated in Ref. 113. For the 3*d*/5*d*-multilayered ferromagnetic metals, the orbital magnetic moment anisotropy in 3*d*-metals cannot completely explain the VCMA effect. In addition to the magnetic moments in 3*d* metals, those in 5*d* metals should be considered in treating the total PMA energy in the system. Moreover, both the orbital magnetic moments and the electric quadrupole mechanisms (second term of Equation (2) and Figure 6b) of Pt dominate the VCMA in the case of *L*1_0_-FePt, as shown in Ref. 116. As discussed in the recent review paper [111], it has been widely recognized that the XAS/XMCD spectroscopy is a powerful tool to investigate the voltage-induced effects in spintronic devices [28,113,116,119,120,121,122,123,124].

A much larger VCMA coefficient can be obtained when compared with that of purely electronic origin if we use a chemical reaction [122,125]. For example, a VCMA coefficient exceeding 10,000 fJ/Vm originating from reversible oxygen ion migration has been demonstrated in the Co/GdO*_x_* system. In Ref. 122, XAS/XMCD spectroscopy at the Co absorption edge was employed to a Ta (4 nm)/Pt (3 nm)/Co (0.9 nm)/GdO*_x_* (33 nm)/Ta (2 nm)/Au (12 nm) multilayer and found that an applied voltage changes the oxidation state and magnetization of the Co. Ref. 125 also reports real-time measurements of such an electrochemical VCMA effect. The operating speed strongly depends on the applied voltage and temperature, which strongly indicates that the electrochemical VCMA requires a thermal activation process. The reported maximum speed was in the sub-millisecond range. Therefore, such large values of the electrochemical VCMA seem attractive, but lie beyond the scope of VCMA studies for working memory applications. A similarly large VCMA effect with limited operating speed has been observed in many systems with electrochemical reactions [28,126,127] and/or charge traps [128,129].

Recently, strain-induced modulation of electronic structures and its influence on the VCMA effect has attracted attention [130,131]. For example, Hibino et al. reported a high VCMA coefficient of +1600 fJ/Vm in a Pt/Co/Pd/MgO structure at 10 K [95]. Here, the thin Pd layer possesses a magnetic moment that is induced by the proximity effect from the adjacent Co layer. At room temperature, a conventional linear VCMA effect with an efficiency of −90 fJ/Vm was observed. On the other hand, at lower temperatures below 100 K, a strong nonlinear VCMA effect appeared with the sign reversal. They explained that the observed effect can be attributed to the temperature dependence of the strain in the Pd. Similarly, Kato et al. reported a VCMA coefficient of over +1000 fJ/Vm at room temperature in an Ir/tetragonal FeCo/MgO structure [132]. So far, only static measurements have been done in these experiments. A demonstration of a high speed response is required to confirm whether they actually originate from the purely-electronic VCMA effect or not.

## 4. Materials Research for a Large VCMA Effect

The VCMA coefficient is one of the most important parameters for the scalability design of voltage-torque MRAM. When the cell size is reduced, we need to increase the PMA of the free layer to maintain the target thermal stability. As described in Section 2, voltage-induced dynamic switching requires the elimination of the PMA during the precessional dynamics.

**Figure 10:**
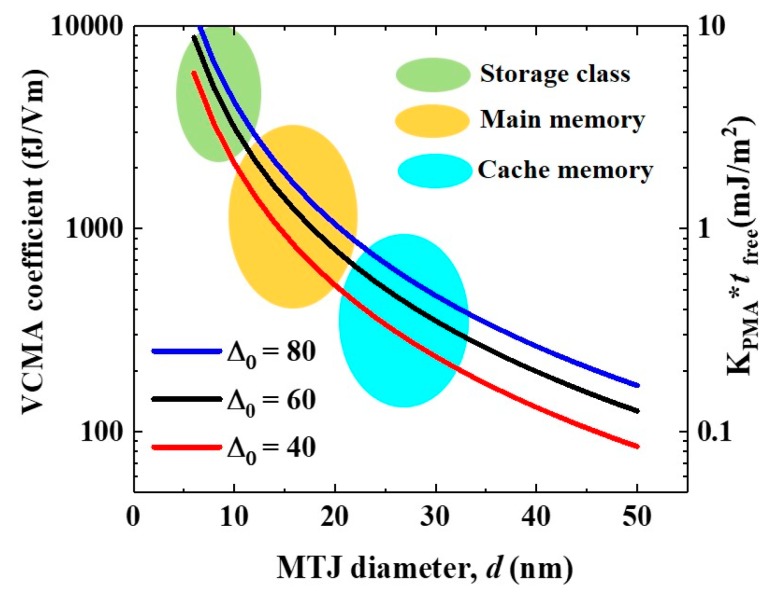
Scalability issue for voltage-torque MRAMs. The dependence of the required *K*_PMA_*t*_free_ and VCMA coefficient on the diameter of the MTJ was estimated for each thermal stability factor (Δ_0_).

Figure 10 shows a simple estimate of the PMA and VCMA coefficient required to consider the scalability [34,35]. As the simplest example, if we assume a free layer whose PMA is only determined by the interface magnetic anisotropy at the interface with the dielectric layer, the effective PMA energy is expressed as

(4) $$K_{\mathrm{PMA}}\left(E\right)=\frac{K_{i}\left(E\right)}{t_{\mathrm{free}}}-\frac{1}{2}\mu_{0}M_{S}^{2}$$

Here, *t*_free_ and *M*_S_ are the thickness and saturation magnetization of the free layer. *K_i_*(*E*) is the PMA under application of the electric-field (*E*), and it is given by

(5) $$K_{i}\left(E\right)=K_{i}\left(E=0\right)-\eta E$$

where *η* is the VCMA coefficient. The thermal stability Δ(*Ε*) of the free layer under the application of the electric-field can be expressed by

(6) $$\Delta \left(E\right)=\frac{K_{\mathrm{PMA}}\left(E\right)At_{\mathrm{free}}}{k_{B}T}=\Delta_{0}-\frac{\eta A}{k_{B}T}E$$

Here, *A* and Δ_0_ are the area of the free layer and the thermal stability under zero electric-field, respectively.

Consequently, the VCMA coefficient, *η*, which is required to eliminate Δ_0_ can be expressed as,

(7) $$\eta =\frac{k_{B}T\Delta_{0}}{AE_{\mathrm{SW}}}$$

where *E*_SW_ is the amplitude of the switching electric-field. 

For the curves in Figure 10, it was assumed that *t*_free_ = 1 nm and *E*_SW_ = 1 V/nm for each value of Δ_0_. If we take cache memory applications as an example, the required *K*_PMA_*t*_free_ values range from 0.2 mJ/m^2^ to 0.5 mJ/m^2^, depending on the target Δ_0_ values; consequently, the required VCMA coefficient is estimated to be from 200 fJ/Vm to 500 fJ/Vm. The main memory applications need higher *K*_PMA_*t*_free_ values in the range from 0.6 mJ/m^2^ to 1.5 mJ/m^2^. As a result, the required VCMA coefficient is in the range from 600 fJ/Vm to 1500 fJ/Vm. However, in experiments that have only focused on the purely-electronic VCMA effect, the achieved VCMA coefficient that is demonstrated in practical materials, such as CoFeB, has been limited to about 100 fJ/Vm [71,78,81,98].

**Figure 11:**
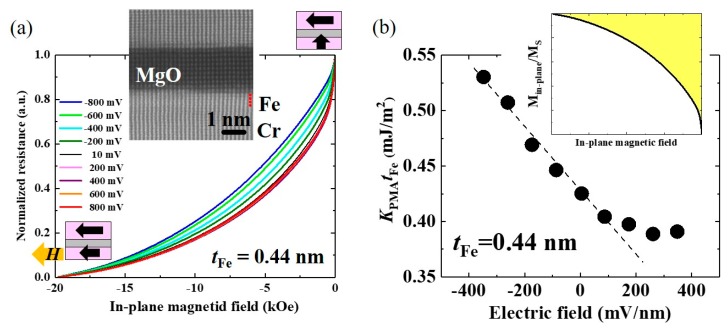
(**a**) Bias voltage dependence of normalized tunnel magnetoresistance (TMR) curves measured under in-plane magnetic fields for an orthogonally magnetized MTJ consisting of Cr/ultrathin Fe (0.44 nm)/MgO/Fe (10 nm). The inset shows a cross-sectional TEM image of the MTJ. (**b**) Applied electric-field dependence of *K*_PMA_*t*_Fe_ values. The inset displays an example of a normalized *M*-*H* curve. *K*_PMA_ was evaluated from the yellow-colored area with the saturation magnetization value that was obtained by a SQUID measurement. Reprinted figure with permission from [133], Copyright 2017 by the American Physical Society.

We employed a fully epitaxial Cr/ultrathin Fe/MgO system as a standard system for the materials research of VCMA effect [133], because large interface magnetic anisotropy can be obtained due to the flat and well-defined Fe/MgO interface [134,135,136] when compared to MTJs with noble metal buffers, which can have the problem of surface segregation [137]. To evaluate the VCMA properties, we used molecular beam epitaxy to prepare orthogonally-magnetized MTJ structures that consisted of a MgO seed (3 nm)/Cr buffer (30 nm)/ultrathin Fe (*t*_Fe_)/MgO (*t*_MgO_ = 2.3 nm)/Fe(10 nm) on MgO(001) substrates. Here, the bottom ultrathin Fe layer is the voltage-controlled free layer with perpendicular magnetic easy axis and the top 10 nm-thick Fe is the in-plane magnetized reference layer. Figure 11a shows an example of the applied bias voltage, *V*_bias_, and dependence of the half-MR loop measured under an in-plane magnetic field, *H*_ex_. The vertical axis is normalized using the maximum (*H_ex_* = 0 Oe) and minimum (*H*_ex_ = −20 kOe) resistances. The Fe thickness is fixed at *t*_Fe_ = 0.44 nm.

**Figure 12:**
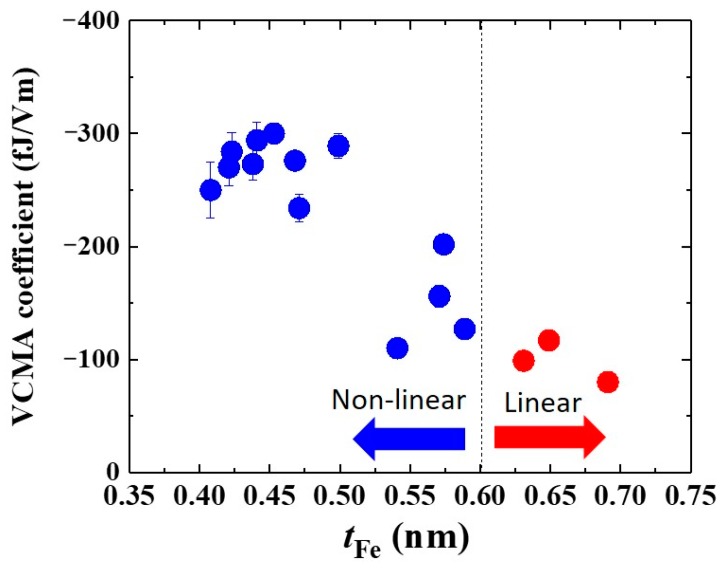
Fe thickness dependence of the VCMA coefficient observed in a Cr/ultrathin Fe(*t*_Fe_)/MgO/Fe structure. A large VCMA coefficient with nonlinear behavior was found in the thinner Fe thickness range, *t*_Fe_ < 0.6 nm (blue dots). Reprinted figure with permission from [133], Copyright by the American Physical Society.

The application of an in-plane magnetic field tilts the magnetization of the ultrathin Fe layer into the magnetic hard axis, while that of the reference layer remains in the film plane (see the drawings in Figure 11a). Therefore, the effective perpendicular anisotropy field is reflected in the saturation behavior of tunneling resistance. The tunneling conductance, *G*, depends on the relative angle (*θ*) between the magnetizations of the free and reference layers, i.e. *G*(*θ*) = *G*_90_ + (*G*_P_−*G*_90_)cos*θ*. Here, *G*_90_ and *G*_P_ are the conductance under the orthogonal and parallel magnetization configurations. Therefore, the ratio of the in-plane component of the magnetization of the free layer, *M*_in-plane_, to its saturation magnetization, *M*_S_, is expressed as

(8) $$\frac{M_{\mathrm{in}-\mathrm{plane}}}{M_{S}}=\cos\theta =\frac{R_{90}-R\left(\theta \right)}{R\left(\theta \right)}\frac{R_{P}}{R_{90}-R_{P}}$$

where *R*_P_ is the MTJ resistance in the parallel magnetization configuration, *R*_90_ is the MTJ resistance in the orthogonal magnetization configuration, and *R*(*θ*) is the MTJ resistance when the magnetization of the ultrathin Fe layer is tilted towards the in-plane direction at angle *θ* under the application of an in-plane magnetic field. Using Equation (8), we can evaluate the normalized in-plane magnetization versus the applied magnetic field. The inset in Figure 11b shows an example of a normalized *M*-*H* curve measured under *V*_bias_ = 10 mV. The PMA energy, *K*_PMA_ can be calculated from *M*_in-plane_ (*H*) with the saturation magnetization value evaluated by SQUID measurements (yellow area in the inset of Figure 11b). Figure 11b summarizes the applied electric-field, *V*_bias_/*t*_MgO_, dependence of *K*_PMA_*t*_Fe_. With ultrathin layers of Fe, an unexpected nonlinear VCMA effect was observed. Under the application of negative voltages, the PMA monotonically increases with a large VCMA coefficient of −290 fJ/Vm. On the other hand, the PMA deviates from a linear relationship under the application of positive voltages. Figure 12 summarizes the Fe thickness dependence of the VCMA coefficient. This nonlinear VCMA effect was only observed with ultrathin layers of Fe, *t*_Fe_ < 0.6 nm (blue dots), and the usual linear VCMA effect appears for thicker layers (red dots). Xiang et al. systematically investigated the tunneling conductance, the PMA, and the VCMA effect in a similar system to determine the origin of the nonlinear VCMA effect, but the MgO was replaced by a MgAl_2_O_4_ barrier, which has smaller lattice mismatch with Fe. Interestingly, they found strong correlation between the VCMA effect and the quantum well states of Δ_1_ band formed in an ultrathin Fe layer that is sandwiched between the Cr and MgO layers [138]. These results may indicate that artificial control of the electronic states in an ultrathin ferromagnetic layer may provide a new approach for designing the VCMA properties. In addition to the influence of quantum well states, we found that intentional Cr doping at the Fe/MgO interface can enhance the PMA and the VCMA effect [62]. Therefore, intermixing with the bottom Cr buffer may also have an influence on the observed large VCMA effect. A theoretical investigation to understand the role of the inter-diffused Cr atoms has been proceeded [139,140].

A large VCMA effect can be obtained with the Cr/ultrathin Fe/MgO system; however, we can only induce an enhancement in the PMA. As explained in Section 2, reduction in the PMA is required for voltage-induced dynamic switching of the perpendicularly-magnetized free layer.

Nakamura et al. proposed inserting a heavy metal monolayer at the Fe/MgO interface to improve the VCMA properties, and found using first-principles calculations that 5*d* transition metals, such as Ir and Os, would be effective in enhancing the VCMA coefficient [141]. A few experimental trials of interface engineering that included the insertion of a heavy metal layer at a CoFe-based film/MgO interface have been reported [81,142]; however, the VCMA coefficient was still less than 100 fJ/Vm. Ir seems to be a promising candidate for this purpose due to its huge spin-orbit coupling constant, which is more than 10 times larger than that of 3*d* transition ferromagnets [141].

**Figure 13:**
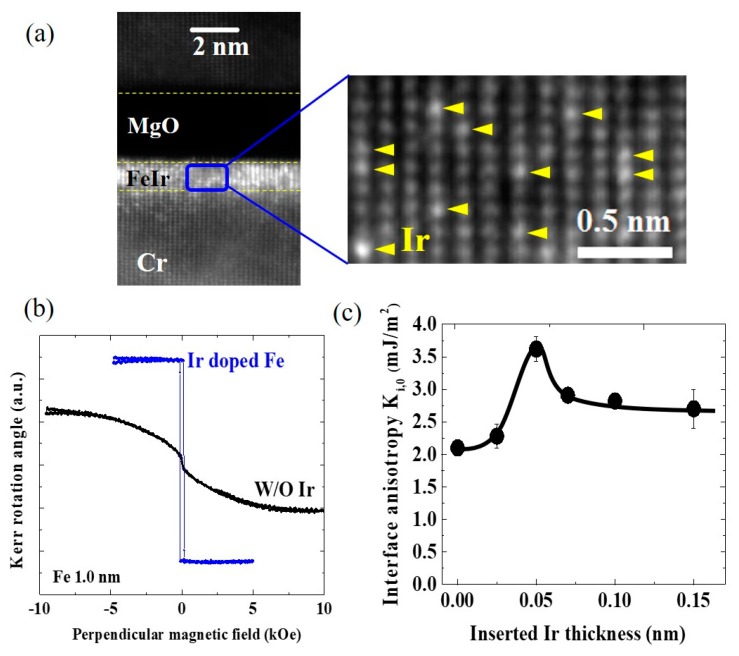
(**a**) HAADF-STEM images of a multilayer structure of Cr/ultrathin Ir-doped Fe/MgO. Inter-diffused Ir atoms can be identified by atomic-scale Z-contrast HAADF-STEM imaging as indicated by the yellow arrows. (**b**) Comparison of the polar MOKE hysteresis curves for pure Fe (1 nm)/MgO and Fe (1 nm)/Ir (0.1 nm)/MgO structures. (**c**) Dependence of the intrinsic interface magnetic anisotropy energy, K_i,0_, on the thickness of the Ir layer. Reproduced from [35]. CC BY 4.0.

We prepared multilayer structures consisting of Cr (30 nm)/ultrathin Fe(*t*_Fe_)/Ir(*t*_Ir_)/MgO (2.5 nm) with indium-tin oxide (ITO) or Fe (10 nm) top electrodes to investigate the impact of the introduction of Ir on the interfacial PMA and the VCMA effect [35]. The ultrathin Ir layer was inserted between the Fe and MgO layers; however, we found that the Ir atoms were dispersed inside the Fe layer during the post-annealing process, as seen in the HAADF-STEM images in Figure 13a. Atomic-scale *Z*-contrast HAADF-STEM imaging enabled the identification of inter-diffused Ir atoms as bright spots that are indicated by yellow arrows. The first-principles calculation predicts strong in-plane anisotropy at the Ir/MgO interface [141]; however, we observed an unexpected enhancement in the PMA. Figure 13b shows a comparison between the polar MOKE hysteresis curves of a single Fe layer (*t*_Fe_ = 1.0 nm) and an Ir-doped Fe layer formed the bilayer structure consisting of Fe (1.0 nm)/Ir (0.1 nm)). The pure Fe layer exhibits large saturation fields of about 7 kOe, which indicated an in-plane magnetic easy axis. On the other hand, the introduction of the quite thin Ir doping layer resulted in transition of the magnetic easy axis from the in-plane to the out-of-plane direction. Figure 13c summarizes the dependence of the intrinsic interfacial magnetic anisotropy, *K*_i,0_, on the thickness of the Ir layer. With appropriate Ir doping, *K*_i,0_ reaches 3.7 mJ/m^2^, which is about 1.8 times that observed at the Fe/MgO interface (2.0 mJ/m^2^) [35,134]. 

**Figure 14:**
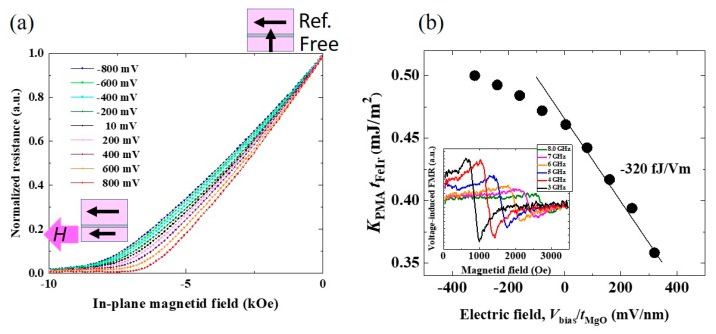
(**a**) Bias voltage dependence of normalized TMR curves measured under in-plane magnetic fields for an orthogonally-magnetized MTJ consisting of Cr/Ir-doped Fe(0.82 nm)/MgO/Fe(10 nm). (**b**) Applied electric-field dependence of *K*_PMA_*t*_FeIr_. The inset shows an example of voltage-induced FMR excitation measured by a homodyne detection technique, which proves the high speed responsiveness of the observed VCMA effect. Reproduced from [35]. CC BY 4.0.

The Ir doping also has an effect on the VCMA. Figure 14a shows an example of the bias voltage effect on the TMR curves that were measured under in-plane magnetic fields for an orthogonally-magnetized MTJ with an Ir-doped Fe free layer (*t*_FeIr_ = 0.82 nm; formed from Fe (0.77 nm)/Ir (0.05 nm)). The saturation field shifts with changes in the applied voltage, as is the case in a pure Fe/MgO structure. However, the applied electric-field dependence of *K*_PMA_*t*_FeIr_ exhibits a completely different trend when compared with that observed in the Fe/MgO structure. We observed a large reduction in PMA with a VCMA coefficient of −320 fJ/Vm under positive voltages (see Figure 14b). It is interesting that such a low doping concentration of Ir, which is even thinner than one monolayer, can have a drastic effect on the VCMA properties. In addition, voltage-induced FMR measurements confirmed the high speed response of the VCMA effect, as shown in the inset in Figure 14b. Thus, the observed large VCMA comes from purely-electronic origin.

**Figure 15:**
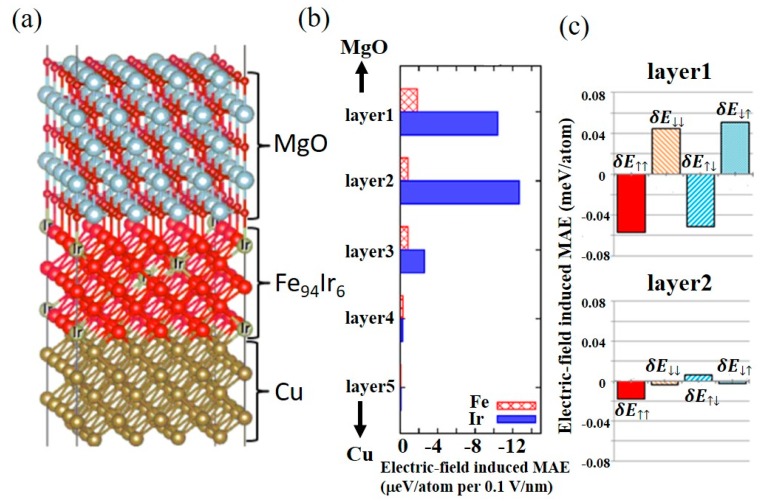
First principles calculations of the electric-field induced magnetic anisotropy energy change in an Ir-doped Fe/MgO system. (**a**) Supercell structure used for the calculation, consisting of MgO (5 ML)/FeIr (5 ML)/MgO (5 ML). (**b**) Atomic-resolved magnetic anisotropy energies (MAE) change induced by an electric-field of 0.1 V/nm in MgO. The Ir concentration was maintained at about 6% in the FeIr layer. (**c**) The electric-field induced MAE arising from second-order perturbation of the spin-orbit coupling for Ir atoms in layers 1 and 2. Reproduced from [35]. CC BY 4.0.

A theoretical analysis using first-principles calculation was performed in Cu(5ML)/Fe_94_Ir_6_(5ML)/MgO(5ML) structures to discuss the physical origin of the large VCMA effect in Ir-doped Fe. The Ir-doped bcc Fe was modeled by a supercell consisting of 4×4 unit cells as shown in Figure 15a. Figure 15b depicts the atomic-resolved electric-field induced magnetic anisotropy energies (MAE) for the Fe and Ir atoms. The variation in the MAE for the Ir atoms is more than five times greater than that for the Fe atoms. Interestingly, MAE change in the second layer (layer 2 in Figure 15b) from the interface with the MgO layer is larger than that of the layer 1, contrary to expectations.

We also attempted to divide the MAE into contributions from the spin-flip and spin-conserved terms between the occupied and unoccupied states. Figure 15c shows the voltage-induced changes in MAE that arise from second-order perturbation of the Ir sites in layers 1 and 2. The electric-field modulation of the spin-conserved term for the majority spin occupied and unoccupied states δE_↑↑_ is larger than that for the minority spin states δE_↓↓_. On the other hand, the spin-flip terms that are by the electric-field, δE_↑↓_ and δE_↓↑_ have almost the same absolute value, but with opposite sign, so the VCMA effect that arises from the spin-flip term is small. Therefore, the large VCMA effect in Ir-doped Fe is mainly caused by the electric-field effect on the majority spin Ir-5*d* states and it can be interpreted by the modulation in the first term of Equation (2), i.e. the orbital magnetic moment mechanism.

**Figure 16:**
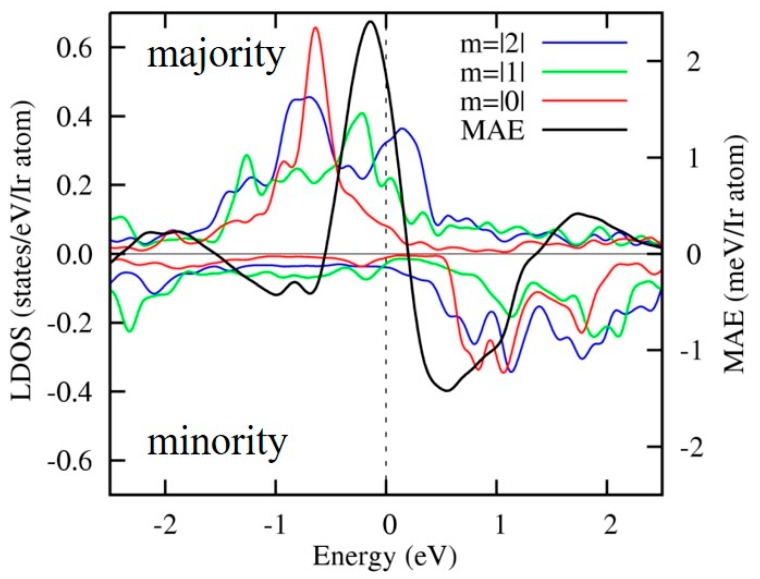
Spin polarized local density of states of Ir-5*d* orbitals and magnetic anisotropy energy as a function of the band energy in layer 2.

Figure 16 shows the density of states for Ir atoms in layer 2. The majority spin 5*d* states are dominant near the Fermi level, since the minority spin 5*d* states near the Fermi level form bonding and anti-bonding states by hybridization with the minority spin Fe-3*d* states. On the other hand, the majority spin 5*d* states are well-localized when compared with the minority spin states near the Fermi level. Figure 16 also shows the MAE as a function of the Fermi energy shift (black line). The PMA energy is drastically modified by a small shift in Fermi energy reflecting the localized majority spin states and the large spin-orbit coupling of the Ir atoms. As a result, a large VCMA is obtained for the charge-doping effect even in layer 2.

The theoretical calculations predict the larger VCMA effect exceeding a few thousand fJ/Vm by inserting a monolayer of Ir at the Fe/MgO interface; however, such a structure can drastically degrade the TMR properties in the MTJ device, in addition to the strong in-plane anisotropy. On the other hand, if Ir doping can improve both the PMA and the VCMA effect while minimizing degradation in TMR, the MTJs should be much more manufacturable, even by sputtering processes. In fact, the enhancement of the PMA and the VCMA effect by Ir doping has also been confirmed in polycrystalline MTJs that are mainly prepared by sputtering [143]. We still have numerous choices for the 4*d* and 5*d* elements, therefore materials engineering using heavy metal doping has enormous possibilities for further improvement in the interfacial PMA and VCMA properties.

## 5. Towards Reliable Voltage-Induced Dynamic Switching

In this section, recent experimental trials for reliable voltage-induced dynamic switching are discussed. As shown in Figure 4, voltage-driven magnetization switching is initiated by precession of the magnetization that is induced by the VCMA effect and the associated voltage-torque, which is proportional to the time derivative of the applied voltage. During the application of a voltage, the magnetization precesses around the effective field while undergoing magnetization damping. Once the voltage is turned off, the magnetic anisotropy immediately recovers as the ferromagnetic layer/dielectric layer junction discharges, and the magnetization relaxes into one of two polarities. We can achieve bipolar magnetization switching using a unipolar voltage pulse with a controlled duration since the polarity of the final state can be controlled by the voltage pulse width. In the absence of thermal fluctuations, the magnetization trajectory during the switching process is uniquely determined for a given initial state and voltage pulse shape, and therefore error-free magnetization switching can be achieved by choosing the appropriate voltage pulse width. However, in practice, the magnetization inevitably suffers thermal fluctuations and that results in the stochastic generation of write errors. Special care must be taken when attempting to reduce the write errors in voltage-torque MRAM cells. In the case of STT, the current polarity determines the polarity of magnetization switching, and a longer pulse may be used to reduce write errors. On the other hand, in the case of voltage-induced dynamic switching, a longer pulse dampens the magnetization along the effective field direction, and this degrades the switching accuracy.

**Figure 17:**
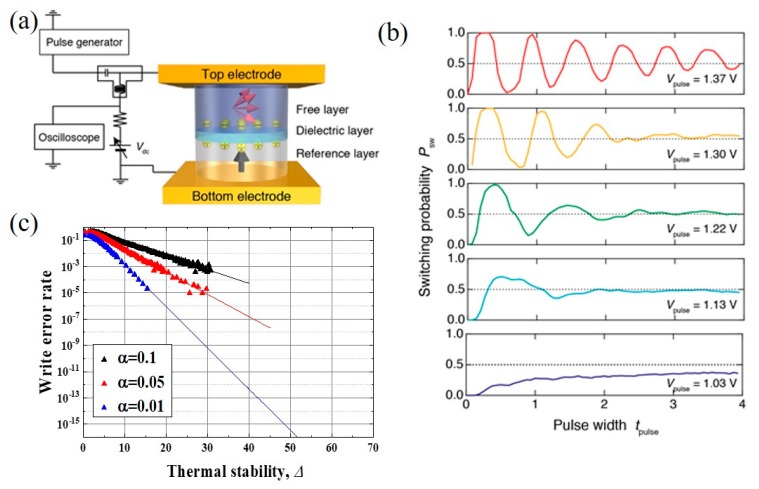
(**a**) Experimental setup for evaluating the WER of an MTJ. (**b**) Pulsed-voltage-driven magnetization switching in a p-MTJ. (**c**) WER as a function of Δ obtained from numerical simulations.

Although earlier experiments have characterized the basics of voltage-driven magnetization switching, it was only in 2016 that the WER in a practical MTJ was quantitatively evaluated for the first time [105]. Figure 17a shows a schematic illustration of an experimental setup for evaluating the WER of an MTJ. Voltage pulses that were generated by the pulse generator are fed to the MTJ and these switch the free layer magnetization. The free layer magnetization direction, either parallel or antiparallel with respect to the reference layer magnetization, can be monitored via the TMR effect. 

Figure 17b displays the typical behavior of voltage-driven magnetization switching; *P*_sw_ is the switching probability, *t*_pulse_ is the pulse width; and, *V*_pulse_ is the voltage amplitude. When *V*_pulse_ is small, the VCMA effect cannot completely eliminate the magnetic energy barrier; therefore, the magnetization switching in this region is dominated by thermal activation. As *V*_pulse_ is increased, well-defined oscillation of *P*_sw_ appears, which is a signature of precession-mediated switching induced by the VCMA effect. As discussed in Section 2, the highest *P*_sw_ is obtained at *t*_pulse_ that corresponds to one-half the magnetization precession cycle, and then *P*_sw_ gradually moves toward 0.5 while undergoing damped oscillations. This behavior can be understood as the combined action of magnetization damping and thermal fluctuations. 

**Figure 18:**
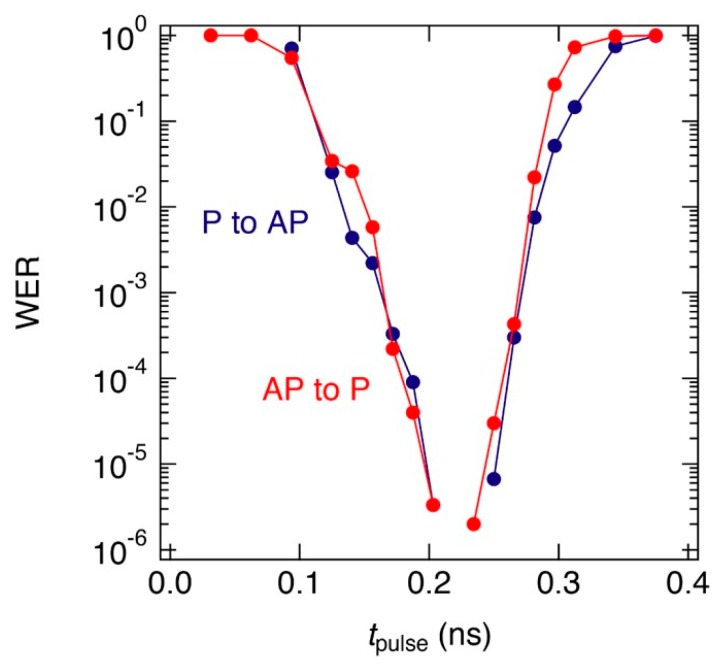
Example of the optimized WER as a function of *t*_pulse_ observed in a perpendicularly-magnetized MTJ consisting of Ta/(Co_50_Fe_50_)_80_B_20_/MgO/reference layer. The blue and red symbols represent the WER of parallel (P) to antiparallel (AP) and AP to P switching, respectively. Reprinted figure with permission from [109], Copyright 2019 by the IOP Publishing Ltd.

In Ref. 105, Shiota et al. employed perpendicularly magnetized MTJ (p-MTJ) that consisted of a reference layer/MgO/Fe_80_B_20_/W cap and experimentally demonstrated a WER of 4 × 10^−3^. They also demonstrated in numerical simulations that the WER could be reduced by improving the thermal stability factor, Δ and by reducing the magnetic damping, α of the free layer, as shown in Figure 17c. An improved Δ effectively reduces the thermal fluctuations in the initial state and in the relaxation process after switching. Moreover, a lower α can reduce the influence of thermal fluctuations during the switching process, which leads to more accurate writing. However, it should be noted that, the larger the value of Δ, the larger the VCMA efficiency required, otherwise the magnetization switching is dominated by thermal activation, and well-controlled magnetization switching cannot be obtained. By using CoFeB/MgO/CoFeB p-MTJs, Grezes et al. experimentally investigated the WER and the read disturbance rate as a function of read/write pulse width and amplitude, and examined the compatibility of the bit-level device performance for integration with CMOS processes [110]. They also simulated the performance of a 256 kbit voltage-torque MRAM block in a 28 nm CMOS process, and showed the capability of the MTJs for delivering WERs below 10^−9^ for 10 ns total write time by introducing the read verify processes. The introduction of read verify processes makes it possible to reduce the effective WER, however it causes an increase in the total writing time. Therefore, we need further effort to reduce the essential WER that is induced by single pulse switching. Recently, Shiota et al. showed that improvement in the PMA and VCMA properties can be achieved in the MTJ consisting of Ta/(Co_30_Fe_70_)_80_B_20_/MgO/reference layer, and demonstrated a WER of 2 × 10^−5^ without the read verify process [106]. Further optimization of the composition of the CoFeB alloy and the device structure allowed for a WER lower than 10^−6^ to be achieved, as shown in Figure 18 [109]. In this case, the introduction of a once read verify process enables a practical WER of the order of 10^−12^.

**Figure 19:**
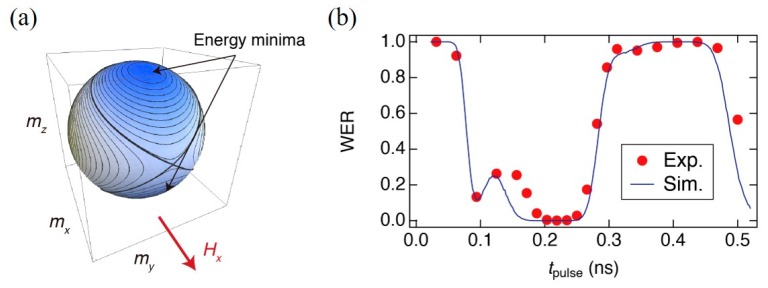
(**a**) Contour plot of energy density in the absence of a bias voltage. (**b**) Appearance of a local peak in the WER observed in an MTJ consisting of Ta/(Co_30_Fe_70_)_80_B_20_ (1.1 nm)/MgO/reference layer. The filled circles and the lines represent experimental data and numerical simulations, respectively. Reprinted figure with permission from [107], Copyright 2018 by the American Physical Society.

In addition to materials engineering, a physical understanding of the voltage-driven magnetization dynamics is also needed in order to facilitate reductions in the WER. Recent studies [107,108] showed that numerical simulations that are based on the macrospin approximation could well reproduce the experimental data by taking into account thermal fluctuations and magnetization damping. In macrospin approximation, the free layer spins are represented by a magnetic moment ***M***, and its time evolution can be obtained by numerically solving the Landau-Lifshitz-Gilbert equation:

(9) $$\frac{dM}{dt}=\gamma M\times H_{\mathrm{eff}}+\frac{\alpha M}{M_{s}}\times \frac{dM}{dt}$$

where *M*_s_ is the saturation magnetization, *t* is the time, α is the damping constant, and ***H***_eff_ is the effective field given by

(10) $$H_{\mathrm{eff}}=-\frac{dE}{dM}$$

and *E* is the energy density expressed as

(11) $$E=K_{\mathrm{PMA}}\left(1-m_{z}^{2}\right)-M_{s}H_{x}m_{x}$$

where ***m*** = (*m_x_*, *m_y_*, *m_z_*) is the magnetization unit vector and *H_x_* is an in-plane bias magnetic field. As displayed in Figure 19a, without the VCMA effect, the magnetization has two energy equilibrium at ${\tilde{m}}_{\pm }=\left({\tilde{m}}_{x},\;0,\;\pm \sqrt{1-{\tilde{m}}_{x}^{2}}\right)$, where ${\tilde{m}}_{x}=M_{s}H_{x}/\left(2K_{\mathrm{PMA}}\right)$, one maximum at *m_x_* = −1, and one saddle point at *m_x_* = 1. By letting *K*_PMA_ fall to zero, the magnetization precesses around *H_x_* associated with damping, and the appropriate duration can switch the magnetization direction. 

Figure 19b displays a typical plot of the dependence of WER on *t*_pulse_ that was observed in an MTJ consisting of a Ta/(Co_30_Fe_70_)_80_B_20_ (1.1 nm)/MgO/reference layer. The amplitude of the in-plane component of the bias magnetic field is 890 Oe. The filled circles and the line denote data were obtained from experiments and numerical simulations, respectively [107]. Good agreement with the experimental data suggests the validity of the model used for the numerical simulations. It is noteworthy that the WER exhibits a local maximum at a certain *t*_pulse_, which cannot be explained just by considering the VCMA effect. A detailed analysis of the magnetization trajectory revealed that thermal agitation during the relaxation process (*i.e.*, after the pulse application) induces the transition of the magnetization between the precession orbits surrounding the energy minima and that the precession-orbit transition enhances the WER. The numerical simulations also revealed that the probability of the precession-orbit transition depends on *t*_pulse_ (see Ref. 107 for more details). In the present case, the probability is maximized at around *t*_pulse_ = 0.12 ns. This results in the appearance of a local maximum in the WER, and it narrows the operating *t*_pulse_ range for which reliable magnetization switching is assured. As the appearance of the WER local maximum is related to magnetization fluctuations during the relaxation process, we need to reduce its influence by improving the PMA and VCMA properties in order to achieve a wide operating *t*_pulse_ range.

**Figure 20:**
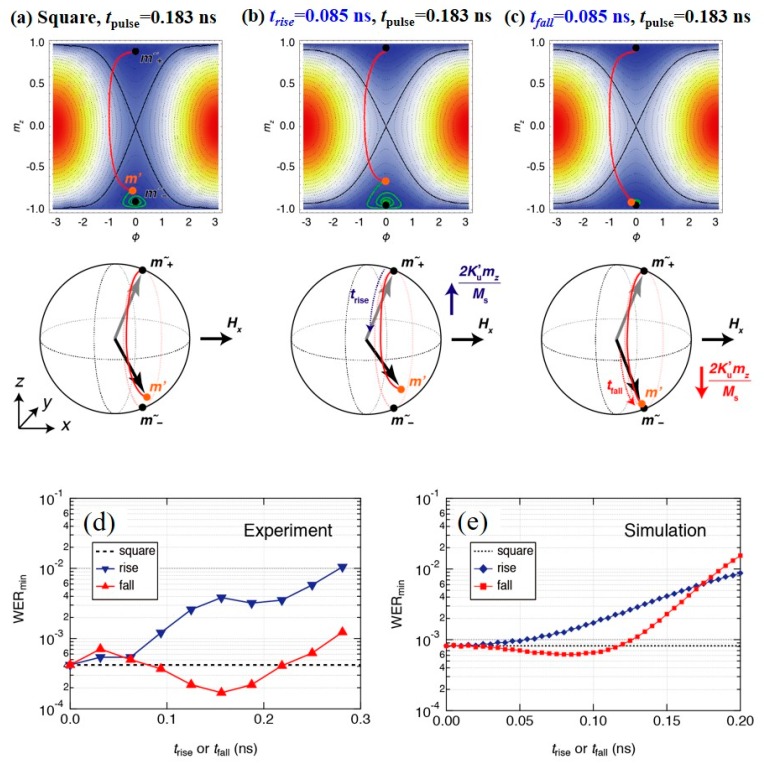
(**a**)–(**c**) Effects of pulse shaping on magnetization trajectory. The red and green lines represent the magnetization trajectory during and after application of the pulse, *t*_pulse_, respectively. (**d**), (**e**) WER minimum as a function of rise time (blue symbols) and fall time (red symbols). (**d**) experimental results; (**e**) numerical simulation results. Reprinted figure with permission from [108], Copyright 2019 by the American Physical Society.

In addition to *t*_pulse_, a recent study revealed that the WER depends in a unique manner on the rise time (*t*_rise_) and fall time (*t*_fall_) [108]. Figure 20 displays the magnetization trajectories that were obtained by using three different waveforms. When a pulsed voltage is applied, the magnetization rotates from *m*+ towards *m*− (red line) and, after the pulse, the magnetization relaxes to either ${\tilde{m}}_{+}$ or ${\tilde{m}}_{-}$, depending on *t*_pulse_ (green line). An important thing is that, due to the nonzero magnetization damping, the magnetization direction at the end of the voltage pulse (*m*′) never reaches ${\tilde{m}}_{+}$ or ${\tilde{m}}_{-}$ whatever *t*_pulse_ is chosen as long as one uses square pulses (Figure 20a). Therefore, it takes some time before the magnetization settles down to the energy minimum. During that time, the magnetization is subjected to thermal agitation, and a finite number of write errors will be counted. When a nonzero *t*_rise_ and/or nonzero *t*_fall_ is introduced, the magnetization is subjected not only to *H_x_*, but also to the anisotropy field due to the uncompensated PMA *K*_PMA_′(*V*,*t*), which is given by

(12) $$H_{\mathrm{ani}}=\;-\frac{2K_{\mathrm{PMA}}^{\prime }\left(V,t\right)m_{z}}{M_{s}}$$

Since the polarity of *H*_ani_ switches according to the polarity of *m_z_*, it applies additional torque to the magnetization that tilts the magnetization to *H_x_* during *t*_rise_ (Figure 20b), and it pulls the magnetization away from *H_x_* during *t*_fall_ (Figure 20c). As a result, for *t*_rise_ = 0.085 ns, *m*′ comes closer to the saddle point, whereas, for *t*_fall_ = 0.085 ns, *m*′ almost overlaps with ${\tilde{m}}_{-}$ and thereby one can minimize the time that is required for relaxation. This suggests that there is a certain *t*_fall_ which can minimize the WER. Indeed, such WER reduction is experimentally obtained and the numerical simulations reproduce it, as shown in Figure 20d,e. 

**Figure 21:**
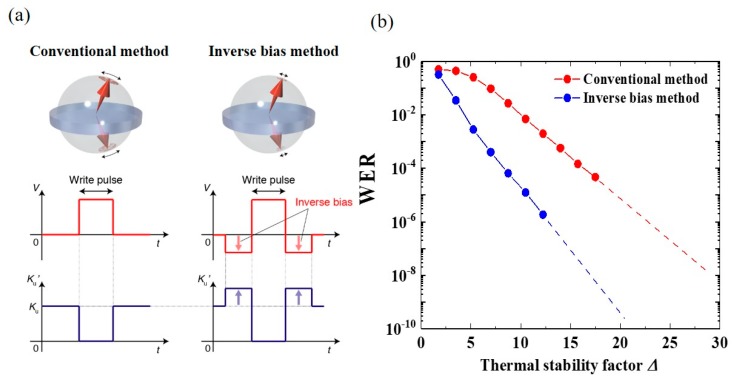
(**a**) Comparison of write pulse sequence in conventional and inverse bias methods. (**b**) Numerically obtained WER as a function of Δ using two different methods.

The inverse bias method is another unique technique for reducing the WER. Figure 21a illustrates the write sequence of the conventional and inverse bias methods. In the inverse bias method, a bias voltage with a negative polarity is applied before and after the write pulse. If the system exhibits a linear VCMA effect, then the inverse bias enhances the *K*_PMA_ of the free layer, and thereby reduces the thermal fluctuations in the initial state and during the relaxation process. It should be noted that inverse biases can also be used for the pre-read and read verify processes, which thereby offers a read-disturbance-free operation as well as WER reduction. Noguchi et al. first proposed the inverse bias method [37] and the effectiveness was later studied using numerical simulations [144]. In Ref. 144, a substantial reduction in WER was confirmed by introducing inverse biases, whose absolute intensity was the same as that of the write pulse, but with opposite sign (see Figure 21b). 

Since precise control of voltage-driven magnetization switching relies on the precise control of the voltage pulse shape, accurate calculation and shaping of the voltage pulse waveform [38,145] are also an important technique for studying the voltage-driven magnetization dynamics in detail. The procedure that is presented in Ref. 145 allows for one to accurately analyze and control the voltage waveform applied to an MTJ. This is especially important in the development of voltage-torque MRAM, because the MTJ resistance becomes much higher than 50 Ω to suppress the flow of charge current, whereas nearly all microwave interconnects have a characteristic impedance of 50 Ω. This impedance mismatch gives rise to multiple reflections between the signal source and the MTJ, and/or the deformation of the waveform, and this obscures the correlation between the applied voltage waveform and the induced magnetization dynamics.

An external bias magnetic field has been used to determine the axis for magnetization precession in most experimental demonstrations of voltage-induced dynamic switching. However, the application of a magnetic field is not suitable for practical circuits. Therefore, we also need efforts to replace the external bias field by an effective field, such as through crystalline anisotropy and exchange bias fields. Matsumoto et al. proposed using a combination of a conical magnetization state and shape anisotropy to induce precessional switching under zero-bias magnetic field [146]. Conical magnetization states have been mainly studied in multilayer structures containing Co, such as Co/Pt and Co/Pd [147,148,149], however recently it can be realized, even in a practical CoFeB/MgO structure [150,151,152]. Therefore, the above proposed structure might be applicable if we can realize a sufficiently-high thermal stability while keeping the conical states.

## 6. Conclusions

Electric-field control of spin has the potential to make substantial impact on the development of novel nonvolatile memory with ultra-low operating power, as well as the expected zero stand-by power. The utilization of the voltage-controlled magnetic anisotropy (VCMA) effect is a promising approach to realizing voltage-torque MRAMs. Bi-stable magnetization switching has been demonstrated while using precessional dynamics that are induced by the VCMA effect. The purely-electronic VCMA effect originates from electric-field induced modification of the electronic structure at the interface between an ultrathin ferromagnet and a dielectric layer, such as MgO. In a 3*d* transition ferromagnet, e.g. Fe and Co, the voltage-induced change in the orbital magnetic moment plays an important role in the origin of the VCMA effect through the carrier accumulation/depletion effect at the interface. On the other hand, in a 3*d*/5*d* composite system, e.g. L1_0_-FePt film, an electric quadrupole mechanism also has significant influence on the VCMA effect. To increase of the VCMA coefficient, the utilization of proximity-induced magnetism in a 5*d* transition metal, which has large spin-orbit coupling, is promising. A large VCMA coefficient of −320 fJ/Vm has been achieved in an Ir-doped ultrathin Fe layer with a demonstration of high-speed responsiveness. As for the reliability of writing while using voltage-induced dynamic switching, low write error rates of the order of 10^−6^ have been realized by improving the thermal stability and the VCMA effect in practical perpendicularly-magnetized MTJs. Further enhancement in the VCMA coefficient is the key to demonstrating the potential for scalability and realizing more reliable switching for voltage-torque MRAM. A novel nonvolatile memory maintaining low operating power as well as zero stand-by power can provide a broader option for the design of memory hierarchy in future data-driven society. We expect that the voltage-torque MRAM has the potential to be applied in IoT edge devices and wearable/implantable computing systems, in which, ultimately, low power consumption is strongly demanded. Furthermore, the voltage-control of spin may also lead to the improvement in other spintronic devices, such as a voltage-tuned magnetic sensor, spin-torque oscillator, and spin-based neuromorphic devices.
